# 

**Published:** 1903-01-17

**Authors:** 


### NOTICE TO CORRESPONDENTS.

All MSS., Letters, Books for Review, and other matters intended for the Editor should be addressed The Editor, "The Hospital," The Hospital Building, 28 & 29 Southampton Street, Strand, W.C.

The Editor cannot undertake to return rejected MS., even when accompanied by stamped directed envelope.

**Notice to Advertisers and Subscribers.**

All Advertisements, Orders for Copies of The Hospital, and Business Communications should be addressed to **THE MANAGER, (Not to the Editor)**. The Hospital, 28 & 29 Southampton Street, Strand London, W.C.

The Cost of Subscription, post free, is as follows.—Per week, 2½d.; per quarter, 2s. 8d.; per half-year, 5s. 3d.; per annum, 10s. 6d. Foreign:—Per annum, 15s. 2d., payable, in all cases, in advance.

**NOTICE.—Vol. XXXII. of "The Hospital" (April to September 1902) in handsome cloth binding is now on sale at the office of this Journal, price, 7s. 6d. Cases for binding the Numbers contained in this Volume 1s. 6d. Index to Vol. XXXII., 2½d., post free.**

**The Monthly Part for January is now ready. Price 6d., by post 8d.**

### THE STUDENT OF MEDICINE.

### APPOINTMENTS.

**G. Barber**, M.R.C.S.Eng., L.R.C.P.Lond., appointed Honorary Ophthalmic Surgeon to the Kalgoorlie Hospital, Western Australia. **Frank Barnes**, M.B., B.S.Lond., F.R.C.S.Eng., appointed Resident Surgical Officer to the General Hospital, Birmingham. **S. Cameron**, M.B., Ch.B., appointed Resident Medical Officer to the Chelsea Hospital for Women. **Alfred Campbell**, F.R.C.S.Edin., L.R.C.P.Lond., appointed Government Medical Officer and Vaccinator at Young, New South Wales. **F. J. Charlton**, M.R.C.S., L.R.C.P.Lond., appointed House Surgeon and Dispenser to the Beverley Dispensary and Hospital. **F. Hobill Cole**, M.B., Ch.B.Melb., appointed Examiner in Pharmacy and Materia Medica in the University of Melbourne. **H. A. Easton**, M.R.C.S., L.R.C.P.Lond., appointed Assistant Medical Superintendent of the Croydon Union Infirmary. **William Elgee**, M.R.C.S.Eng., L.R.C.P.Lond., appointed Health Officer for Bellevue, Western Australia. **G. B. Flux**, M.D.Brux., M.R.C.S., L.R.C.P., L.S.A., appointed Assistant Anæsthetist at King's College Hospital. **Edward A. Graham**, M.B., B.Ch.Melb., appointed Government Medical Officer and Vaccinator at Deniliquin, New South Wales. **W. S. Holmes**, M.B., Ch.B.Vict., appointed Junior House Surgeon to the Oldham Infirmary. **John Howe**, M.B., Ch.B.Vict., appointed Medical Officer and Public Vaccinator to the District of Levenshulme and part of West Gorton (No. 8) of the Chorlton Union, Manchester. **H. J. Hutchens**, M.R.C.S.Eng., L.R.C.P.Lond., D.S.O., appointed Assistant Bacteriologist to the West Riding of Yorkshire County Council. **S. H. McCoy**, M.B., B.A.Toronto, appointed Clinical Assistant to the Chelsea Hospital for Women. **S. P. Matthews**, M.R.C.S., L.R.C.P.Lond., appointed Medical Officer and Public Vaccinator for the No. 3 District of the Horsham Union. **R. O. Moon**, M.A., M.D., M.R.C.P., appointed Physician to Out-patients at the Infirmary for Consumption, Margaret Street, W. **J. M. Morris**, M.A., M.B., C.M.Edin., appointed Medical Officer of Health for the Borough of Neath. **G. S. Murray**, M.B., Ch.B.Edin., appointed Senior House Surgeon to the Oldham Infirmary. **W. A. Murray**, M.B., Ch.B.Edin., appointed Deputy Principal Medical Officer for the Gold Coast Colony. **H. R. Oswald**, M.D.Edin., appointed Coroner for the South Eastern District of London. **George Pernet**, M.R.C.S.Eng., L.R.C.P.Lond., appointed Assistant to the Skin Department of University College Hospital. **Sidney Plowman**, F.R.C.S.Eng., L.R.C.P.Lond., appointed Examiner in Pharmacy and Materia Medica in the University of Melbourne. **Stanley J. D. Read**, M.B., appointed Public Vaccinator for the Northern District and Officer of Health for the Borough of Raywood, Victoria. **W. G. Savage**, B.Sc.Lond, M.D., D.P.H., appointed Medical Officer of Health for the Borough of Colchester. **G. Crewdson Thomas**, M.D., C.M., M.R.C.P., appointed Physician to Out-patients at the Infirmary for Consumption, Margaret Street, W. **Derwent H. R. Waldron**, M.B. C.M.Edin., appointed Senior Medical Officer of the Gold Coast Colony. **C. F. Wightman**, F.R.C.S.Eng., L.R.C.P.Lond., appointed District Medical Officer of the Royston Union.

### Useful Books for Nurses' Libraries.

### THE NURSING PROFESSION: HOW AND WHERE TO TRAIN.

Being a Guide to Training for the Calling of a Nurse.

Edited by Sir HENRY BURDETT, K.C.B.

Crown 8vo., 2|- net, 2/4 post free.

### A HANDBOOK FOR NURSES.

By J. K. WATSON, M.D., M.B., C.M.

New and Revised Edition.

Crown 8vo., profusely illustrated, cloth gilt.

5/- net, 5/4 post free.

### NURSING: ITS THEORY AND PRACTICE.

Being a complete Text-book of Medical, Surgical, and Monthly Nursing.

By PERCY C. LEWIS, M.D., M.R.C.S., L.S.A.

New Edition, Enlarged and Revised (17th thousand), profusely illustrated with over 100 new Cuts. Crown 8vo., cloth gilt.

3/6 post free.

### NURSING: ITS PRINCIPLES AND PRACTICE.

By ISABEL ADAMS HAMPTON.

New and Enlarged Edition.

Crown 8vo., illustrated, cloth gilt.

7/6 net, 7/10 post free.

### SURGICAL WARD-WORK AND NURSING.

By ALEXANDER MILES, M.D.Edin., C.M., F.R.C.S.E.

Second Edition, Enlarged and entirely Revised.

Demy 8vo., copiously illustrated, cloth gilt.

3/6 net, 3/10 post free.

### ELEMENTARY ANATOMY AND SURGERY FOR NURSES.

By WILLIAM MCADAM ECCLES, M.S.Lond., M.B., F.R.C.S., L.R.C.P.

Crown 8vo., profusely illustrated,

Cloth. 2/6 post free.

### ELEMENTARY PHYSIOLOGY FOR NURSES.

By C. F. MARSHALL, M.D., B.Sc., F.R.C.S.

Crown 8vo., illustrated.

Cloth. 2/- post free.

### THE NURSE'S DICTIONARY OF MEDICAL TERMS AND NURSING TREATMENT.

Compiled for the Use of Nurses.

By HONNOR MORTEN.

Fourth and Revised Edition (50th thousand) Demy 16mo. (suitable for the apron pocket).

Bound in cloth boards, 160 pp., 2/- post free.

In handsome leather gilt, 2/6 net, 2/8 post free.

### HOSPITAL SISTERS AND THEIR DUTIES.

By EVA C. S. LÜCKES, Matron, London Hospital.

Third Edition, enlarged and partly re-written.

Crown 8vo., about 200 pp. cloth gilt.

2|6 post free.

### OPHTHALMIC NURSING.

By SYDNEY STEPHENSON, M.B., F.R.C.S.E.

New and Revised Edition.

Crown 8vo., profusely illustrated with Original Drawings, List of Instruments required, and a Glossary of Terms. Cloth.

3/6 net, 3/10 post free.

### ART OF MASSAGE.

By MRS. CREIGHTON HALE.

Second Edition.

Demy 8vo., 150 pp., profusely illustrated with over 70 Special Cuts.

Cloth gilt,

6/- post free.

### A PRACTICAL HANDBOOK OF MIDWIFERY.

By FRANCIS W. HAULTAIN, M.D.

Now Ready.

New and Revised Edition.

Profusely illustrated with Original Cuts, Tables, &c.

Handsomely bound in leather.

6/- net, 6/3 post free.

*Of all Booksellers, or of*

THE SCIENTIFIC PRESS, LTD., 28 & 29 Southampton Street, Strand, London, W.C.

